# The Influence of Gentrification on Adverse Birth Outcomes in California

**DOI:** 10.1007/s11524-024-00902-7

**Published:** 2024-09-27

**Authors:** Xing Gao, Mahasin S. Mujahid, Amani M. Nuru-Jeter, Rachel Morello-Frosch

**Affiliations:** 1 Department of Obstetrics, Gynecology & Reproductive Sciences, University of California San Francisco, San Francisco, CA USA; 2 Division of Epidemiology, School of Public Health, University of California Berkeley, Berkeley, CA USA; 3 Division of Community Health Sciences, School of Public Health, University of California Berkeley, Berkeley, CA USA; 4 Division of Environmental Health Sciences, School of Public Health, University of California Berkeley, Berkeley, CA USA; 5 Department of Environmental Science, Policy and Management, University of California Berkeley, 130 Mulford Hall, Berkeley, CA 94720 USA

**Keywords:** Neighborhood environment, Birth outcome, Racial inequities, Gentrification, Social epidemiology

## Abstract

Evidence has documented the effects of place on perinatal outcomes, but less is known about the sociopolitical mechanisms, such as gentrification, that shape neighborhood context and produce spatialized inequities in adverse birth outcomes. Leveraging a diverse sample in California, we assessed the associations between gentrification and birth outcomes: preterm birth, small-for-gestational-age, and low birth weight. Gentrification was measured using the Freeman method and the Displacement and Gentrification Typology. Descriptive analysis assessed outcome prevalence and race and ethnicity distribution by exposure and participant characteristics. Overall and race and ethnicity-stratified mixed effects logistic models examined associations between gentrification and birth outcomes, sequentially adjusting for sociodemographic status and pregnancy factors, with a random intercept to account for clustering by census tract. In a sample of 5,116,131 births, outcome prevalence ranged from 1.0% for very preterm birth, 5.0% for low birth weight, 7.9% for preterm birth, and 9.4% for small-for-gestational-age. Adjusting for individual-level factors, gentrification was associated with increased odds of preterm birth (Freeman OR = 1.09, 95% CI 1.07–1.10; Displacement and Gentrification Typology OR = 1.11, 95% CI 1.09–1.13). While Displacement and Gentrification Typology–measured gentrification was consistently associated with greater odds of adverse outcomes, Freeman-measured gentrification was associated with slightly lower odds of small-for-gestational-age and low birth weight. Furthermore, gentrification was associated with birth outcome odds across multiple racial and ethnic groups, but the directions and magnitudes of the associations varied depending on the gentrification assessment methodology and the outcome assessed. Results demonstrate that gentrification plays a role in shaping adverse birth outcomes in California.

**Supplementary Information:**

The online version contains supplementary material available at 10.1007/s11524-024-00902-7.

## Introduction

In the United States, there are substantial racial and ethnic inequities in adverse birth outcomes, which influence infant mortality and quality of life. In 2020, non-Hispanic Black pregnant people experienced the highest rate of delivering a baby preterm or with low birth weight (LBW), which were 1.5 times and twice as high, respectively, compared to rates among non-Hispanic White pregnant people [1]. Rates of preterm birth (PTB) and LBW were also elevated among Indigenous and Hispanic pregnant people [1]. Infants who are born preterm and with LBW face higher risks for early-onset chronic conditions, such as diabetes and cardiovascular diseases, as well as future pregnancy outcomes, resulting in the intergenerational transmission of health inequities [2]. A comprehensive understanding of the multi-level causes, ranging from individual-level, contextual, and structural factors, can inform efforts to address the enduring inequities in adverse birth outcomes.

A robust body of literature has documented the effect of neighborhood environments, including the built environment, community socioeconomic resources, and social environment, on birth outcomes [3, 4]. Overall, these studies found that health-harmful neighborhood characteristics are associated with increased risk of PTB, LBW, and small-for-gestational-age (SGA). Furthermore, the influence of the neighborhood environment may be stronger among racially marginalized populations [5, 6]. Past and contemporary policies and programs, such as historical redlining, housing discrimination, and exclusionary zoning, have continuously shaped neighborhood conditions by distributing (dis)investment along racial and class lines [7]. For example, extant studies have reported that neighborhood segregation, which may impact residents’ access to educational, economic, and healthcare resources, is associated with increased risk of PTB and LBW, particularly among Black birthing people and their infants [6]. Neighborhood conditions can undergo rapid changes related to gentrification, a process that also interacts with other mechanisms of neighborhood-level investment and deprivation such as racial and economic segregation.

Gentrification describes the racialized and profit-driven process in which a disinvested neighborhood with lower socioeconomic status and real estate value, as well as a higher concentration of racially and economically marginalized residents, experiences an influx of private sector- and government-led development tailored for higher-income, White individuals moving into the neighborhood [8, 9, 10]. Gentrification may improve physical amenities, such as green space, transit, healthy food retailers, and housing quality, which can be protective against adverse birth outcomes [11, 12]. However, access to these resources may be limited by privatization and commodification, making them unaffordable or unavailable to low-income long-term residents [13]. Gentrification may also result in increased exposure to psychosocial stressors due to displacement, disintegration of community support and social networks, and increased financial strain in the face of rising housing costs, leading to adverse birth outcomes [13, 14].

Gentrification, as situated within the system of racial capitalism that extracts socioeconomic value from racially marginalized groups, may be a mechanism through which neighborhoods and populations with a history of segregation and exclusion experience further exploitation and displacement [8, 13, 15]. The effects of gentrification on birth outcomes may be differential across racial and ethnic groups. For example, while studies have documented that early-stage gentrification may parallel crime reduction, the increase in punitive policing that accompanies gentrification-related neighborhood development may be especially harmful to Black and other racially marginalized pregnant people [16]. Two previous studies have assessed the associations between gentrification and adverse birth outcomes and reported mixed findings [17, 18]. Differences in the definition of gentrification and its empirical operationalization may explain the mixed findings across studies.

This paper investigated the influence of neighborhood gentrification on adverse birth outcomes, including PTB, SGA, and LBW, among California births from 2005 to 2017. Gentrification was measured using two assessment methodologies: the Freeman method and the Displacement and Gentrification Typology [19, 20]. Using race and ethnicity as markers for differential exposure to exploitation and devaluation under racial capitalism, we examined the associations between gentrification and birth outcomes in the overall population and within five racial and ethnic groups [21].

## Methods

### Study Population

This study leveraged a population-based state-wide sample of all births in California between 2005 and 2017, using birth certificate data from the Department of Health Care Access and Information. This dataset included information on the characteristics of birthing people and their infants: health and sociodemographic factors, perinatal outcomes, and address at the time of delivery. Addresses were geocoded to link to census tract identifiers, which enabled linkage to neighborhood-level gentrification variables.

From a total sample of 6,738,539 births, we excluded births if they could not be linked to a census tract, were missing, or had implausible gestational age (< 22 weeks or > 45 weeks), implausible pregnant person age (< 10 or > 60 years old), implausible birth weight (< 100 g or > 9000 g), or were plural births. We also excluded births that were missing complete exposure, outcome, or covariate information, and births where the birthing person’s race did not meet inclusion criteria (Supplemental Fig. 1). The final dataset included 5,116,131 births in 7575 census tracts; the mean number of births per tract was 675.4, with a minimum of 1 and a maximum of 6386. Study protocols were approved by the California Committee for the Protection of Human Subjects and the Institutional Review Boards of UC Berkeley (Protocol number: 13–05-1231).

### Study Outcome

Preterm birth (PTB) was defined as births after 24 weeks and before 37 weeks of gestation, and very preterm birth (VPTB) was defined as births after 24 weeks and before 32 weeks of gestation. Small-for-gestational-age (SGA) births had a birth weight less than the United States sex-specific tenth percentile of weight for each week of gestation [22]. Lastly, we assessed low birth weight (LBW) cases as infants born weighing less than 2500 g. As a sensitivity analysis, we also examined birth weight continuously, using birth weight *z*-scores for all infants and term birth weight for infants born between 37 and 44 weeks of gestation [22].

### Gentrification

Neighborhoods were defined as census tracts. Metropolitan and Micropolitan Statistical Areas, defined by the Office of Management and Budget, were used as the regional boundaries, which we linked to census tracts using the Federal Information Processing System codes. Tract characteristics were compared with the corresponding regional characteristics.

We measured changes across two 10-year periods: 2000–2010, characterized using the 2000 Decennial Census and the 2008–2012 American Community Survey (ACS) 5-Year Estimates; 2007–2017, measured using the 2005–2009 and 2015–2019 ACS 5-Year Estimates [23]. Births were linked to their respective periods based on year, with a 5-year lag between the start of the gentrification period and the birth year to maximize the likelihood that the neighborhood was experiencing gentrification when the birth occurred (Supplemental Table 1).

#### Freeman Method

Using census data, the Freeman method classified gentrification based on socioeconomic indicators [14, 19]. Tracts were classified as eligible for gentrification if 50% of the census blocks in the tract were urban, and the median household income and proportion of housing built in the prior two decades were lower than or equal to the regional median. Otherwise, the tract was determined to be ineligible for gentrification, or “excluded.” Among the tracts eligible for gentrification, those that saw an increase in median home value and percentage of residents with a bachelor’s degree that was larger than the corresponding regional change in these two characteristics during the respective period were classified as gentrifying, and the rest were classified as not gentrifying. In summary, this method classified census tracts as eligible for gentrification and gentrifying, eligible for gentrification and not gentrifying, and ineligible for gentrification. Tracts classified as “eligible for gentrification and not gentrifying” were used as the referent group to be comparable to existing findings on gentrification [18]. We also conducted a sensitivity analysis with “ineligible for gentrification” or excluded as the referent group to enable comparison to conceptually similar referent group using the Displacement and Gentrification Typology.

#### Displacement and Gentrification (D&G) Typology

The D&G Typology leveraged census data and the Zillow Home Value Index to classify neighborhoods into nine categories based on community income and housing affordability. The specific criteria for each category are described in Supplemental Table 2. For this analysis, we further collapsed the nine categories into three broad stages of neighborhood change: (1) displacement, which included the categories “Low Income/Susceptible to displacement” and “Ongoing displacement of low-income households”; (2) gentrification, which included the categories “At risk of gentrification,” “Early ongoing gentrification,” and “Advanced gentrification”; and (3) exclusive, which included the categories “Stable moderate/Mixed income,” “At risk of becoming exclusive,” “Becoming exclusive,” and “Stable/Advance exclusive.” Tracts classified as “Exclusive” were used as the referent group. We made a small modification to the “Becoming Exclusive” category by excluding the criterion on the in-migration rate due to the lack of data availability. Materials on this measure can be found at https://github.com/urban-displacement/displacement-typologies.

Comparing the two measures, the Freeman method and its variations have been the most commonly used in epidemiologic studies [24, 25]. This method evaluates multiple socioeconomic and housing features of the neighborhood. The D&G Typology, on the other hand, was developed as a Neighborhood Early Warning System to identify patterns of investment and sociodemographic changes. It emphasizes housing affordability for low- and middle-income families and considers spatial proximity to increasing housing costs. It also measures displacement, allowing a more nuanced definition of neighborhood changes in addition to gentrification [26].

### Covariates

The sociodemographic covariates from birth certificate data included the pregnant person’s age (years) and the principal source of payment at delivery (private, public, uninsured or other). Pregnancy-related factors included parity (any or no prior live births) and receiving adequate prenatal care, which was assessed using the Kotelchuk index (inadequate or intermediate versus adequate or adequate +) [27].

We used self-reported information on birth certificates to determine the pregnant person’s race and ethnicity. The categories were non-Hispanic (NH) Black, NH Asian/Pacific Islander (API), NH American Indian/Alaska Native (AIAN), NH White, and Hispanic. We did not include pregnant people whose race was reported as “Other” or mixed race due to small sample sizes which may be insufficient for stratified analyses. This analysis conceptualized the variable of race and ethnicity as a proxy measure for exposure to past and present social marginalization that racialized people experience, which may influence how they experience gentrification [21].

### Statistical Analysis

Descriptive analysis assessed the prevalence of birth outcomes by neighborhood gentrification status and individual sociodemographic characteristics. We also examined the distribution of gentrification status and participant characteristics overall and by race and ethnicity.

We used mixed-effects logistic regression models, with a random intercept to account for individuals clustering within neighborhoods, to assess associations between gentrification and birth outcomes. Model 1 adjusted for sociodemographic factors (age and insurance type), which may be confounders by influencing people’s residential location and birth outcomes. Model 2 additionally adjusted for pregnancy-related factors (parity and prenatal care), which may be confounders by influencing neighborhood selection or mediators through which gentrification affects birth outcomes. Sensitivity analysis used mixed-effects linear models to assess associations with birth weight z-score and term birth weight.

Based on prior knowledge that gentrification may affect groups differently based on social marginalization and from assessing interaction terms between exposure and race/ethnicity (*P*-value < 0.001 for all birth outcomes), we used race and ethnicity-stratified models to investigate whether the influence of gentrification varied across racial and ethnic groups.

## Results

Of the 5,116,131 births in the final analytic sample, 7.9% were born preterm, and 1.0% were born very preterm. A total of 9.4% were SGA and 5.0% had LBW. The study sample was 28.0% White, 5.4% Black, 14.8% API, 51.5% Hispanic, and 0.3% AIAN. Table 1 displays the prevalence of adverse birth outcomes by gentrification status and individual sociodemographic and pregnancy-related factors. Individuals who were Black or AIAN, younger than 20, had public insurance, primiparous, and received adequate care were more likely to have experienced adverse birth outcomes.

**Table 1:** Prevalence of adverse birth outcomes by participant characteristics, California, 2005–2017 (*N* = 5,116,131)

	Overall sample *N* (%)	Preterm birth (prevalence %)	Very preterm birth (prevalence %)	Small-for-gestational-age (prevalence %)	Low birth weight (prevalence %)
*N*	5,116,131	7.9	1.0	9.4	5.0
Freeman					
Gentrifiable and not gentrifying	1,281,542 (25.0)	8.6	1.2	9.9	5.4
Excluded	3,504,203 (68.5)	7.6	1.0	9.2	4.8
Gentrifiable and gentrifying	330,386 (6.5)	8.5	1.2	9.4	5.1
Displacement and gentrification typology					
Exclusive	3,237,000 (63.3)	7.4	0.9	9.0	4.7
Displacement	1,589,556 (31.1)	8.8	1.2	10.0	5.4
Gentrifying	289,575 (5.7)	8.7	1.2	10.2	5.4
Race and ethnicity					
Black	275,984 (5.4)	11.4	2.2	14.6	9.2
American Indian/Alaskan Native	14,922 (0.3)	9.5	1.3	8.2	5.2
Asian and Pacific Islander	757,849 (14.8)	7.4	0.8	13.0	5.9
Hispanic	2,632,518 (51.5)	8.5	1.1	9.0	4.9
White	1,434,858 (28.0)	6.3	0.7	7.1	3.8
Age					
< 20	360,488 (7.0)	9.6	1.5	13.0	6.2
20–34	3,770,622 (73.7)	7.4	0.9	9.3	4.7
≥ 35	985,021 (19.3)	9.2	1.2	8.4	5.7
Payment type at delivery					
Public	2,466,967 (48.2)	8.8	1.2	10.0	5.4
Private	2,438,553 (47.7)	7.1	0.9	8.8	4.7
Uninsured/other	210,611 (4.1)	6.7	0.9	9.4	4.4
Primiparous					
Yes	2,024,554 (39.6)	7.6	1.1	12.1	6.0
No	3,091,577 (60.4)	8.0	1.0	7.6	4.3
Adequate prenatal care					
Yes	3,987,548 (77.9)	8.5	1.1	9.0	5.3
No	1,128,583 (22.1)	5.7	0.7	10.7	4.0

Overall distribution is displayed by count and column percentage in parenthesis; birth outcome prevalence by participant characteristics is displayed by percentage

Table 2 shows that the proportion of individuals living in a gentrifying census tract was 6.5% using the Freeman measure, and 5.7% using the D&G Typology measure. The D&G Typology classified 30.9% of the sample as living in neighborhoods undergoing displacement. Furthermore, which census tracts were classified as gentrifying varied across both methods, with 10.2% and 19.3% overlaps (Supplemental Table 2). Black individuals were more likely to live in gentrifying neighborhoods across both exposure assessment methodologies, followed by American Indian/Alaskan Native individuals who were more likely to live in Freeman gentrifying tracts, and Hispanic individuals who were more likely to live in D&G Typology gentrifying tracts.

**Table 2:** Participant characteristics by race and ethnicity, California, 2005–2017 (*N* = 5,116,131)

	Overall	Black	American Indian and Alaskan Native	Asian and Pacific Islander	Hispanic	White
*N*		275,984	14,922	757,849	2,632,518	1,434,858
Freeman						
Gentrifiable and not gentrifying	1,281,542 (25.0)	33.8	22.0	16.1	32.7	14.0
Excluded	3,504,203 (68.5)	57.2	69.6	78.9	60.7	79.4
Gentrifiable and gentrifying	330,386 (6.5)	8.9	8.3	5.0	6.5	6.6
Displacement and gentrification typology						
Exclusive	3,237,000 (63.3)	45.3	64.9	77.3	51.2	81.5
Displacement	1,589,556 (31.1)	47.1	30.5	17.8	41.3	16.2
Gentrifying	289,575 (5.7)	7.6	4.6	4.9	7.5	2.4
Preterm birth	402,873 (7.9)	11.4	9.5	7.4	8.5	6.3
Very preterm birth	52,437 (1.0)	2.2	1.3	0.8	1.1	0.7
Small-for-gestational-age	480,432 (9.4)	14.6	8.2	13.0	9.0	7.1
Low birth weight	255,492 (5.0)	9.2	5.2	5.9	4.9	3.8
Age						
< 20	360,488 (7.0)	10.6	9.6	1.3	10.5	3.0
20–34	3,770,622 (73.7)	74.9	77.3	69.7	75.0	73.1
≥ 35	985,021 (19.3)	14.5	13.2	29.0	14.4	23.9
Payment type at delivery						
Public	2,466,967 (48.2)	60.0	58.8	22.9	67.0	24.8
Private	2,438,553 (47.7)	36.7	38.0	66.8	30.2	71.8
Uninsured/other	21,0611 (4.1)	3.4	3.2	10.3	2.8	3.4
Primiparous						
Yes	2,024,554 (39.6)	40.2	34.8	46.3	34.6	45.0
No	3,091,577 (60.4)	59.8	65.2	53.7	65.4	55.0
Adequate care						
Yes	3,987,548 (77.9)	72.2	69.0	79.5	76.3	81.2
No	1,128,583 (22.1)	27.8	31.0	20.5	23.7	18.8

Overall distribution is displayed by count and percentage in parenthesis; distribution by race and ethnicity is displayed by column percentage

Independent of sociodemographic and pregnancy-related factors, residing in a Freeman gentrifying tract was associated with 1.09 times greater odds of PTB (95% CI 1.07–1.10), but with slightly reduced odds of SGA (OR = 0.96, 95% CI 0.94–0.97) and LBW (OR = 0.96, 95% CI 0.94–0.98). Associations between Freeman gentrification and VPTB were null. In comparison, D&G Typology gentrification was consistently associated with greater odds of all four birth outcomes, with the strongest association observed for LBW (OR = 1.13, 95% CI 1.10–1.16). Displacement was also consistently associated with greater odds of adverse birth outcomes, ranging from an OR of 1.08 for SGA (95% CI 1.07–1.09) to an OR of 1.21 for VPTB (95% CI 1.18–1.23) (Table 3).

**Table 3:** Adjusted odd ratios of adverse birth outcomes associated with gentrification, California, 2005–2017 (*N* = 5,116,131)

	Preterm birth		Very preterm birth		Small-for-gestational-age		Low birth weight	
	Model 1 OR (95% CI)	Model 2 OR (95% CI)	Model 1 OR (95% CI)	Model 2 OR (95% CI)	Model 1 OR (95% CI)	Model 2 OR (95% CI)	Model 1 OR (95% CI)	Model 2 OR (95% CI)
Freeman								
Gentrifiable and not gentrifying	–	–	–	–	–	–	–	–
Excluded	0.94 (0.93–0.95)	0.94 (0.93–0.95)	0.85 (0.83–0.87)	0.84 (0.82–0.86)	0.97 (0.96–0.98)	0.96 (0.95–0.96)	0.91 (0.90–0.93)	0.91 (0.89–0.92)
Gentrifiable and gentrifying	1.08 (1.06–1.10)	1.09 (1.07–1.10)	1.02 (0.98–1.06)	1.02 (0.98–1.06)	0.97 (0.95–0.98)	0.96 (0.94–0.97)	0.96 (0.94–0.98)	0.96 (0.94–0.98)
Displacement and gentrification typology								
Exclusive	–	–	–	–	–	–	–	–
Displacement	1.15 (1.14–1.16)	1.16 (1.15–1.17)	1.26 (1.23–1.29)	1.21 (1.18–1.23)	1.05 (1.04–1.06)	1.08 (1.07–1.09)	1.12 (1.11–1.14)	1.16 (1.14–1.17)
Gentrifying	1.10 (1.08–1.12)	1.11 (1.09–1.13)	1.17 (1.13–1.22)	1.10 (1.06–1.15)	1.08 (1.06–1.10)	1.09 (1.07–1.11)	1.11 (1.09–1.14)	1.13 (1.10–1.16)

Model 1: adjusted for age and insurance type at delivery

Model 2: adjusted for age, insurance type at delivery, parity, and adequate prenatal care

**Fig. 1:**
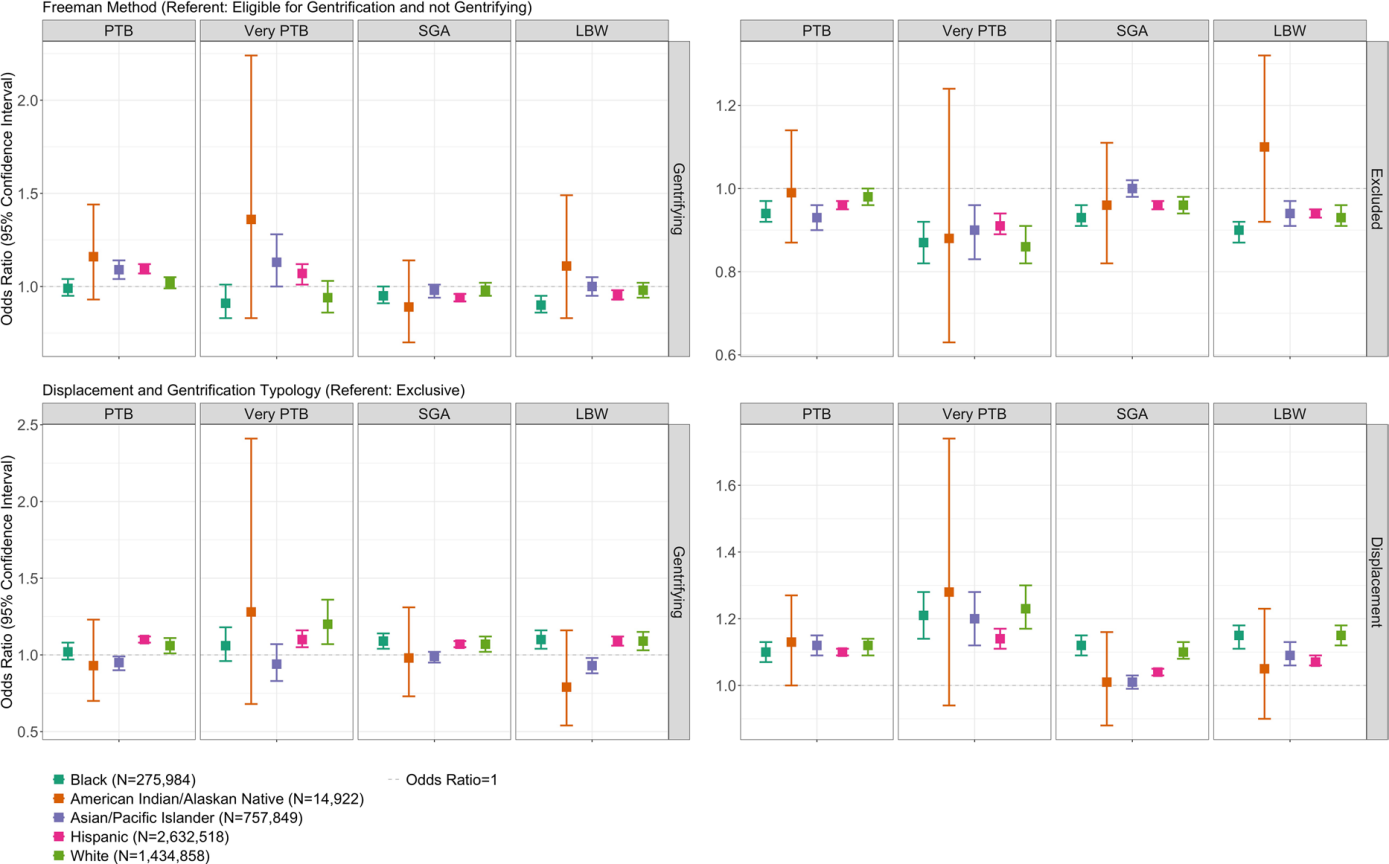
Gentrification and adverse birth outcomes, by race and ethnicity, California, 2005–2017. PTB, preterm birth; SGA, small-for-gestational-age; LBW, low birth weight

Results from race and ethnicity-stratified models, adjusted for sociodemographic and pregnancy-related factors, are shown in Table 4 and Fig. 1. Assessing Freeman gentrification, the overall association with increased odds of PTB and VPTB is driven by associations among API and Hispanic individuals. Among Hispanic individuals, Freeman gentrification was associated with decreased odds of SGA (OR 0.94, 95% CI 0.92–0.96). Freeman gentrification’s associations with reduced odds of LBW were observed among Black and Hispanic individuals. Furthermore, though the confidence intervals were large among AIAN individuals due to the smaller sample size, we observed similar estimates of increased odds of PTB, VPTB, and LBW and reduced odds of SGA.

**Table 4:** Adjusted odd ratios of adverse birth outcomes by gentrification and race and ethnicity, California, 2005–2017 (*N* = 5,116,131)

	Preterm birth				
	Black (*N* = 275,984)	American Indian/Alaskan Native (*N* = 14,922)	Asian/Pacific Islander (*N* = 757,849)	Hispanic (*N* = 2,632,518)	White (*N* = 1,434,858)
Freeman					
Excluded	0.94 (0.92–0.97)	0.99 (0.87–1.14)	0.93 (0.90–0.96)	0.96 (0.95–0.97)	0.98 (0.96–1.00)
Gentrifying	0.99 (0.95–1.04)	1.16 (0.93–1.44)	1.09 (1.04–1.14)	1.09 (1.07–1.12)	1.02 (0.99–1.05)
Displacement and gentrification typology					
Displacement	1.10 (1.07–1.13)	1.13 (1.00–1.27)	1.12 (1.09–1.15)	1.10 (1.09–1.11)	1.12 (1.09–1.14)
Gentrifying	1.02 (0.97–1.08)	0.93 (0.70–1.23)	0.95 (0.90–0.99)	1.10 (1.08–1.12)	1.06 (1.01–1.11)
	Very preterm birth				
	Black (*N* = 275,984)	American Indian/Alaskan Native (*N* = 14,922)	Asian/Pacific Islander (*N* = 757,849)	Hispanic (*N* = 2,632,518)	White (*N* = 1,434,858)
Freeman					
Excluded	0.87 (0.82–0.92)	0.88 (0.63–1.24)	0.90 (0.83–0.96)	0.91 (0.89–0.94)	0.86 (0.82–0.91)
Gentrifying	0.91 (0.83–1.01)	1.36 (0.83–2.24)	1.13 (1.00–1.28)	1.07 (1.01–1.12)	0.94 (0.86–1.03)
Displacement and gentrification typology					
Displacement	1.21 (1.14–1.28)	1.28 (0.94–1.74)	1.20 (1.12–1.28)	1.14 (1.11–1.17)	1.23 (1.17–1.30)
Gentrifying	1.06 (0.96–1.18)	1.28 (0.68–2.41)	0.94 (0.83–1.07)	1.10 (1.05–1.16)	1.20 (1.07–1.36)
	Small-for-gestational-age				
	Black (*N* = 275,984)	American Indian/Alaskan Native (*N* = 14,922)	Asian/Pacific Islander (*N* = 757,849)	Hispanic (*N* = 2,632,518)	White (*N* = 1,434,858)
Freeman					
Excluded	0.93 (0.91–0.96)	0.96 (0.82–1.11)	1.00 (0.98–1.02)	0.96 (0.95–0.97)	0.96 (0.94–0.98)
Gentrifying	0.95 (0.91–1.00)	0.89 (0.70–1.14)	0.98 (0.94–1.01)	0.94 (0.92–0.96)	0.98 (0.95–1.02)
Displacement and gentrification typology					
Displacement	1.12 (1.09–1.15)	1.01 (0.88–1.16)	1.01 (0.99–1.03)	1.04 (1.03–1.05)	1.10 (1.08–1.13)
Gentrifying	1.09 (1.04–1.14)	0.98 (0.73–1.31)	0.99 (0.95–1.02)	1.07 (1.05–1.09)	1.07 (1.02–1.12)
	Low birth weight				
	Black (*N* = 275,984)	American Indian/Alaskan Native (*N* = 14,922)	Asian/Pacific Islander (*N* = 757,849)	Hispanic (*N* = 2,632,518)	White (*N* = 1,434,858)
Freeman					
Excluded	0.90 (0.87–0.92)	1.10 (0.92–1.32)	0.94 (0.91–0.97)	0.94 (0.93–0.95)	0.93 (0.91–0.96)
Gentrifying	0.90 (0.86–0.95)	1.11 (0.83–1.49)	1.00 (0.95–1.05)	0.95 (0.93–0.98)	0.98 (0.94–1.02)
Displacement and gentrification typology					
Displacement	1.15 (1.11–1.18)	1.05 (0.90–1.23)	1.09 (1.06–1.13)	1.07 (1.06–1.09)	1.15 (1.12–1.18)
Gentrifying	1.10 (1.04–1.16)	0.79 (0.54–1.16)	0.93 (0.88–0.98)	1.09 (1.06–1.12)	1.09 (1.03–1.15)

Displaying odds ratios estimates and 95% confidence intervals in parentheses

Model adjusted for age, insurance type, parity, and adequate prenatal care

Freeman referent group: eligible for gentrification and not gentrifying; Displacement and Gentrification Typology referent group: exclusive

Interaction terms between all birth outcomes x race and ethnicity categories were statistically significant (*P-*value < 0.001)

Examining D&G Typology gentrification, associations with increased odds of all four birth outcomes were statistically significant among Hispanic and White individuals, and association with increased odds of SGA and LBW was also observed among Black individuals (SGA OR = 1.09, 95% CI 1.04–1.14; LBW OR = 1.10, 95% CI 1.04–1.16). In contrast, Typology gentrification was associated with reduced odds of PTB (OR = 0.95, 95% CI 0.90–0.99) and LBW (OR = 0.93, 95% CI 0.88–0.98) among API pregnant people. Lastly, Typology displacement was consistently associated with adverse birth outcomes across all racial and ethnic groups, and the magnitude of association was the largest among API individuals for PTB (OR = 1.12, 95% CI 1.09–1.15), among White individuals for VPTB (OR = 1.23, 95% CI 1.17–1.30) and LBW (OR = 1.15, 95% CI 1.12–1.18), and among Black individuals for SGA (OR = 1.11, 95% CI 1.08–1.14). We also found a large magnitude of association, though not significant, between Typology displacement with PTB (OR = 1.13, 95% CI 1.00–1.27) and VPTB (OR 1.28, 95% CI 0.68–2.41) among AIAN people.

Sensitivity analysis assessing continuous birth weight measures found that Freeman gentrification was associated with 0.05 higher birth weight *z*-score (95% CI = 0.04–0.06) and 11.54 g higher term birth weight (95% CI 9.13–13.95) (Supplemental Table 4). D&G Typology gentrification was associated with a 0.03 decrease in birth weight *z*-score (95% CI − 0.04, − 0.03) and 16.71 g lower birth weight (95% CI − 19.89, − 13.53). Using the “Excluded” category as the referent for Freeman gentrification, instead of "Gentrifiable and not gentrifying" as the referent, produced similar results for ORs associated with “Gentrifying” for preterm birth outcomes, and the magnitudes of associations were larger, but associations with SGA became null, and ORs for LBW reversed direction while still remaining modest (Supplemental Table 5).

## Discussion

Leveraging data from a large population-based sample of all births in California between 2005 and 2017, this study investigated the influence of neighborhood-level gentrification and displacement on birth outcomes. We compared findings across two exposure assessment methodologies used to measure gentrification: the Freeman method, a widely utilized measure that leverages socioeconomic and housing information from the census, and the Displacement and Gentrification (D&G) Typology, which underscores housing affordability to low- and middle-income households as a key feature of gentrification. Overall, findings showed that the directions of association varied between the two measures. While D&G Typology gentrification was consistently associated with increased odds of all four birth outcomes, Freeman gentrification had a more mixed pattern of influence on birth outcomes; specifically, this measure was associated with increased odds of preterm birth, but decreased odds of SGA and LBW. Race and ethnicity-stratified models showed that while gentrification mattered for birth outcomes for all groups, the directions and magnitudes of associations varied across exposure assessments and specific birth outcomes. Notably, displacement, as measured by the D&G Typology, revealed the strongest and most consistent positive associations overall, across all adverse birth outcomes, as well as in models stratified by race and ethnicity.

Findings from this study add to existing evidence documenting mixed associations between gentrification and birth outcomes. A study in New York found that gentrification was not associated with PTB in the overall study sample [17], which contradicted our findings documenting the harmful influence of gentrification on PTB using both exposure measures. These divergent findings may be explained by the different geographic locations the analyses and differences in exposure assessment methodologies. A more recent study in California showed that socioeconomic gentrification was protective against PTB, LBW, and SGA, which aligned with our findings for Freeman gentrification in relation to SGA and LBW, but not PTB [18]. Findings from the Beck et al. study contradicted our findings for D&G Typology gentrification, where we found gentrification was associated with increased odds of adverse birth outcomes. Beck et al. used Ding’s measure of gentrification, which shares similarities with the Freeman measure, suggesting that this type of measure may better capture a neighborhood’s upward trajectory but may not reflect some of the negative elements, such as the threat of displacement [18]. These findings highlight the importance of gentrification measurement methodology in capturing the relevant positive and negative influence of gentrification. Furthermore, the lack of consistency in classifying tracts as gentrifying across both the Freeman and D&G methodologies emphasizes the importance of selecting exposure measures based on the conceptualization of gentrification and specifying the aspects that may be most salient to birth outcomes.

Gentrification, occurring within the system of racial capitalism, may impact groups differently based on their positions on the racial hierarchy [8, 15]. Groups that have been racially marginalized may experience exploitation and value extraction to accumulate profit for the more privileged groups. Black, Indigenous, and other people of color may be impacted by the more harmful aspects of gentrification, such as displacement, disintegration of their communities, and rising housing costs, while simultaneously being less able to access newly available resources. Gentrification was most frequently significantly associated with birth outcomes for Hispanic pregnant people. D&G Typology gentrification and displacement were associated with greater odds of all adverse birth outcomes, suggesting that housing affordability to low- and middle-income households may be especially impactful for Hispanic populations. Freeman gentrification yielded more mixed results among Hispanic individuals, consistent with existing literature documenting the heterogenous health effects of gentrification. Latinx communities in the U.S. are navigating and contesting gentrification, highlighting the need to better understand the health consequences of this process [28, 29].

Among Black individuals, D&G Typology gentrification was associated with increased odds of SGA and LBW, and Freeman gentrification was associated with decreased odds of SGA. In a previous study, gentrification was associated with greater odds of PTB only among Black individuals, which was consistent with our results using the D&G Typology measure, but not with findings using the Freeman measure [17]. Evidence has documented the effects of gentrification on Black communities, illuminating the importance of addressing gentrification-related negative health consequences, especially given the inequitably high rates of adverse birth outcomes among this group [17, 30]. Findings among the API group were the only set of results that contradicted findings among the overall sample; D&G Typology gentrification was associated with increased odds of PTB in the overall sample, and the direction of association was in the opposite direction among API individuals. However, Freeman gentrification was associated with higher odds of PTB among API people, yielding mixed results. Studies about the health effects of gentrification on API communities are limited, an important gap given that Asian enclaves are grappling with shrinking size and gentrification-related development in the U.S. [31].

Although our analysis of gentrification yielded mixed results, the D&G Typology displacement was consistently associated with an increased risk of adverse birth outcomes, aligning with studies documenting how housing instability or eviction can have negative consequences for birthing people [32, 33]. Displacement of low-income households due to rising housing costs may hold both individual-level consequences, such as housing instability and financial strain, increasing psychosocial stress and disrupting access to resources, and community-level impact, including the degradation of the social fabric, loss of sense of belonging, and deteriorating community resources [13]. Though our analysis had a smaller sample size of AIAN people, resulting in wide confidence intervals, the estimated odds of VPTB associated with D&G Typology displacement were large. This finding aligns with evidence that Indigenous communities living in urban areas are contending with the housing crisis, rental discrimination, and dispossession, highlighting the urgent need to address the health consequences of this process for AIAN pregnant people [34, 35].

The strengths of this study include the utilization of a state-wide population-based sample offering racial and ethnic diversity and large geographic coverage, comparison of two gentrification assessment measures, assessment of multiple adverse birth outcomes, and inclusion of individual-level confounders in regression modeling. There are also several limitations. First, our study design did not allow the establishment of temporality between the exposure assignment and outcome. However, we implemented a lag between the gentrification measurement years and the birth years, potentially ensuring that the measured change was underway when the birth occurred. Another limitation is the inability to distinguish between the effects of gentrification on long-term residents who are displaced, long-term residents who remain, and new residents who are moving in. Future studies can leverage a longitudinal study design to address these gaps. Lastly, given the elevated risk of adverse birth outcomes among AIAN pregnant people, future studies should leverage larger population-based datasets or data focused on this population to investigate the health consequences of gentrification. More studies are also needed to investigate whether the consequences of gentrification differ for various birth outcomes and to elucidate the mechanisms through which gentrification may affect birth outcomes positively or negatively.

This study demonstrates the impact of gentrification on birth outcomes. The comparison of two gentrification measurement methods highlights the importance of identifying and measuring aspects of gentrification most salient to birth outcomes, including housing affordability and concomitant displacement threats. With an equity lens, findings from this study support future efforts to investigate and address gentrification’s influence on racially marginalized populations who may be especially vulnerable to the consequences of neighborhood upheaval and dispossession.

## Supplementary Information

Below is the link to the electronic supplementary material.

Supplementary file1 (DOCX 100 KB)

## Data Availability

The authors do not have permission to share data. Birth data can be requested from the California Department of Health Care Access and Information.
