# Supplementary material for: The Influence of Gentrification on Adverse Birth Outcomes in California
Source: J Urban Health. 2024 Sep 27;101(6):1143–54. doi: 10.1007/s11524-024-00902-7 (PMC11652705; doi:10.1007/s11524-024-00902-7)
Supplement: Supplementary file 1 — Supplementary file1 (DOCX 100 KB) [file 11524_2024_902_MOESM1_ESM.docx]

Supplemental Table 1: Linkage of gentrification exposure measures with births in California, 2005-2017

|  | Exposure Data Year | | Linked birth years |
| --- | --- | --- | --- |
|  | Beginning of the period | End of the period |  |
| Period 1 | Decennial Census 2000 | American Community Survey 5-Year Estimate: 2008-2012 | Births between 2005 and 2011 |
| Period 2 | American Community Survey 5-Year Estimate: 2005-2009 | American Community Survey 5-Year Estimate: 2015-2019 | Births between 2012 and 2017 |

Supplemental Table 2: Urban Displacement Project’s Displacement and Gentrification Typology Exposure Assessment Methods

| Category | | CRITERIA |
| --- | --- | --- |
| Displacement | LOW INCOME/SUSCEPTIBLE TO DISPLACEMENT | - Low or mixed low-income tract by the end of the period |
|  | ONGOING DISPLACEMENT OF LOW-INCOME HOUSEHOLDS | - Low or mixed low-income tract by the end of the period - Absolute loss of low-income households during the period |
| Gentrification | AT RISK OF GENTRIFICATION | - Low income or mixed-low income tract by the end of the period - Housing affordable to low or mixed-low income households by the end of the period - Did not gentrify during 1990-2000 or during the period - Marginal change in housing costs OR Zillow home or rental value increases in the 90th percentile during the period - Local and nearby increases in rent were greater than the regional median during the period OR the end of period rent gap is greater than the regional median rent gap |
|  | EARLY ONGOING GENTRIFICATION | - Low income or mixed-low income tract by the end of the period - Housing affordable to moderate or mixed moderate-income households by the end of the period - Increase or rapid increase in housing costs OR above regional median change in Zillow home or rental values during the period - Gentrified between 1990-2000 or during the period |
|  | ADVANCED GENTRIFICATION | - Moderate, mixed moderate, mixed high, high income tract by the end of the period - Housing affordable to middle, high, mixed moderate, and mixed high-income households by the end of the period - Marginal change or increase in housing cost - Gentrified between 1990-2000 or during the period |
| Exclusive | STABLE MODERATE/MIXED INCOME | - Moderate, mixed-moderate, mixed-high, high income tract by the end of the period |
|  | AT RISK OF BECOMING EXCLUSIVE | - Moderate, mixed-moderate, mixed-high, high income tract by the end of the period - Housing affordable to middle, high, mixed-moderate, and mixed-high income households by the end of the period - Marginal change or increase in housing cost |
|  | BECOMING EXCLUSIVE | - Moderate, mixed-moderate, mixed-high, high income tract by the end of the period - Housing affordable to middle, high, mixed-moderate, and mixed-high income households by the end of the period - Rapid increase in housing costs - Absolute loss of low-income households during the period - Absolute loss of low-income households during the period - Median income higher at the end of the period compared to the beginning |
|  | STABLE/ADVANCED EXCLUSIVE | - High-income tract in at the beginning of the period - Affordable to high income household by the end of the period - Marginal change, increase, or rapid increase in housing costs |

* We made a small modification to the “Becoming Exclusive” category by excluding the criterion on in-migration rate due to the lack of data availability.

Supplemental Table 3: Comparison of Three Gentrification Classifications of Census Tracts in California, 2005-2017 (N=7,575)

|  |  | Freeman | | | Displacement and Gentrification Typology | | |
| --- | --- | --- | --- | --- | --- | --- | --- |
|  |  | Excluded | Not Gentrifying | Gentrifying | Exclusive | Displacement | Gentrification |
| Freeman | Not Gentrifying (N=1,442) | -- | -- | -- | 35.4% | 54.1% | 10.5% |
|  | Excluded (N=5,404) | -- | -- | -- | 78.8% | 18.2% | 2.9% |
|  | Gentrifying (N=729) | -- | -- | -- | 50.5% | 39.4% | 10.2% |
| Displacement & Gentrification Typology | Exclusive (N=5,139) | 82.9% | 9.9% | 7.2% | -- | -- | -- |
|  | Displacement (N=2,053) | 48.0% | 38.0% | 14.0% | -- | -- | -- |
|  | Gentrification (N=383) | 41.3% | 39.4% | 19.3% | -- | -- | -- |

Displayed by row percentage

Supplemental Table 4: Mean Difference in Continuous Birth Weight Outcomes Associated with Gentrification, by Race and Ethnicity, California, 2005-2017 4

|  |  | Birth Weight Z-Score | | | | | | | | | | |
| --- | --- | --- | --- | --- | --- | --- | --- | --- | --- | --- | --- | --- |
|  | Overall  (N=5,116,131) | | Black  (N=275,984) | | American Indian/Alaskan Native (N=14,922) | | | Asian/Pacific Islander  (N=757,849) | | Hispanic  (N=2,632,518) | | White  (N=1,434,858) |
| **Freeman** |  | |  | |  | | |  | |  | |  |
| Excluded | 0.01  (0.01, 0.01) | | 0.02  (0.01, 0.04) | | -0.01  (-0.06, 0.05) | | | -0.01  (-0.02, -0.00) | | 0.01  (0.00, 0.01) | | 0.00  (0.00, 0.01) |
| Gentrifying | 0.05  (0.04, 0.06) | | 0.04  (0.02, 0.05) | | 0.03  (-0.06, 0.12) | | | 0.01  (0.01, 0.03) | | 0.06  (0.05, 0.06) | | 0.03  (0.02, 0.04) |
| **Displacement & Gentrification Typology** |  | |  | |  | | |  | |  | |  |
| Displacement | 0.00  (-0.01, 0.00) | | -0.02  (-0.03, -0.01) | | 0.03  (-0.03, 0.07) | | | 0.02  (0.01, 0.02) | | 0.00  (0.00, 0.01) | | 0.00  (-0.01, 0.01) |
| Gentrifying | -0.03  (-0.04. -0.03) | | -0.04  (-0.06, -0.02) | | 0.05  (-0.05, 0.16) | | | 0.00  (-0.01, 0.01) | | -0.03  (-0.03, -0.02) | | -0.02  (-0.03, 0.00) |
|  |  | Term Birth Weight (gram) | | | | | | | | | | |
|  | Overall  (N=4,700,956) | | | Black  (N=243,964) | | American Indian/Alaskan Native  (N=13,437) | Asian/Pacific Islander  (N=700,903) | | Hispanic  (N=2,402,088) | | White  (N=1,340,564) | |
| **Freeman** |  | | |  | |  |  | |  | |  | |
| Excluded | 5.31  (3.59, 7.04) | | | 9.65  (4.56, 14.73) | | -4.10  (-26.37, 18.18) | -2.16  (-5.80, 1.49) | | 5.00  (3.15, 6.85) | | -0.53  (-3.56, 2.50) | |
| Gentrifying | 11.54  (9.13, 13.95) | | | 6.37  (-1.53, 14.27) | | -1.30  (-36.48, 33.89) | 3.32  (-2.74, 9.19) | | 15.45  (12.41, 18.45) | | 10.67  (6.40, 14.93) | |
| **Displacement & Gentrification Typology** |  | | |  | |  |  | |  | |  | |
| Displacement | -7.18  (-9.00, -5.37) | | | -14.66  (-19.64, -9.67) | | -0.15  (-20.81, 20.49) | 1.05  (-2.56, 4.67) | | -3.95  (-5.84, -2.06) | | -2.88  (-5.87, 0.11) | |
| Gentrifying | -16.71  (-19.89, -13.53) | | | -15.52  (-24.45, -6.59) | | -11.47  (-54.16, 31.23) | 0.05  (-6.38, 6.49) | | -16.99  (-20.34, -13.64) | | -8.76  (-15.11, -2.42) | |

Displaying odds ratios estimates and 95% confidence intervals in parentheses.

Model adjusted for age, insurance type, parity, and adequate prenatal care

Freeman referent group: eligible for gentrification and not gentrifying; Displacement and Gentrification Typology referent group: Exclusive

Supplemental Table 5: Adjusted Odd Ratios of Adverse Birth Outcomes Associated with Gentrification, California, 2005-2017 (N=5,116,131)

|  | Preterm Birth | | Very Preterm Birth | | Small-for-Gestational-Age | | Low Birth Weight | |
| --- | --- | --- | --- | --- | --- | --- | --- | --- |
|  | Model 1 OR  (95% CI) | Model 2 OR  (95% CI) | Model 1 OR  (95% CI) | Model 2 OR  (95% CI) | Model 1 OR  (95% CI) | Model 2 OR  (95% CI) | Model 1 OR  (95% CI) | Model 2 OR  (95% CI) |
| **Freeman** |  |  |  |  |  |  |  |  |
| Excluded | -- | -- | -- | -- | -- | -- | -- | -- |
| Not Gentrifying | 1.06  (1.05-1.08) | 1.06  (1.05-1.08) | 1.18  (1.15-1.20) | 1.19  (1.16-1.21) | 1.04  (1.03-1.05) | 1.05  (1.04-1.06) | 1.09  (1.08-1.11) | 1.11  (1.09-1.12) |
| Gentrifying | 1.15  (1.13-1.17) | 1.16  (1.14-1.17) | 1.20  (1.16-1.25) | 1.21  (1.17-1.26) | 1.00  (0.98-1.02) | 1.00  (0.99-1.02) | 1.05  (1.03-1.07) | 1.06  (1.04-1.08) |

Model 1: adjusted for age and insurance type at delivery

Model 2: adjusted for age, insurance type at delivery, parity, and adequate prenatal care

Supplemental Figure 1: Analytic Sample Selection, California, 2005-2017
